# January 1st, 1847

**Published:** 1847-01

**Authors:** 


*January* 1*st*, 1847.—On New-Year's Day, when the salutation "Happy New-Year!" is on every lip, we have sought retirement from the noisy hilarity which characterizes this anniversary, to hold in our quiet editorial chair a few words of converse with our readers. We would greet them, one and all, with 'a happy new-year,' not as a mere form, or empty compliment, but as an expression of cordial interest in their welfare. In tendering our 'wishes,' the thought occurs to us, 'what anticipations, at the opening of a new-year, are peculiar to the medical practitioner!' Does he look forward to new developments in life's experience, which will give a new aspect to his aspirations and destiny? Does he venture to hope that before another new-year's day returns, unexpected events may transpire, by which he may be fortunate beyond present rational calculations? Does he flatter himself that by some unlooked-for accident he may become in that period eminent, or rich, or attain to a degree of success exceeding what he may have already realized? Few, we imagine, permit themselves to entertain such reveries. The medical profession affords little scope for the imagination to erect baseless fabrics of visions like these. It is a sober calling, abounding in stern, monotonous realities, with few temptations of world-distinguished honor, or profit, and quite barren in events calculated to excite the creative efforts of a lively fancy. To the medical practitioner the duties of the year which is past, are identical with those which he is prepared to expect for the year which is to come. He does not look for change. Provided he has the satisfaction of reflecting that these duties have been well performed, he has no new leaves to turn over; each annual chapter of his professional career is a repetition of that which preceded it—and so on to the conclusion of the volume of life's history. But has he no bright anticipations; is there nothing to encourage him to persevere; does it follow that because the current of his life flows on in so even a tenor that it is replenished with no springs of peculiar enjoyment? Not so. Indeed, in the very qualities just referred to, is there not cause for congratulation? If our profession furnish few occasions of stirring excitement, and striking incidents, it is free from many of the disappointments and anxieties which are inseparable from more eventful pursuits. If it be without glittering distinctions, it has its own rewards which are more substantial and satisfactory. If it preclude the accumulation of wealth, it enables us to perceive that the amount of splendid misery in the world far exceeds the unhappiness of poverty, and that the best exemplications of health and happiness are among those whose desires are the most moderate. These comparisons not inopportunely enter into the reflections which this day suggests. Every one is apt to feel that his particular calling is singularly oppressive in its annoyances and trials. As medical men we are perhaps too much in the habit of cultivating this sentiment among ourselves, and often those around us are disposed to confirm it by their assent, and kindly proferred sympathy. True, we have our peculiar hardships, arising from the painful character of many of our duties, the irregularity of our labors, the unsparing and ill-timed urgency of their requisitions, and the often uncheering returns with which our best exertion are reciprocated. But we should not shut out of view the many redeeming traits which belong to the exercise of our profession. Do we not cultivate a science which unites with its intrinsic interest motives of the highest philanthropy? Is it not our mission to do good to man; and are we not blessed with the warm friendship of a multitude of grateful hearts? If labor be an indispensible condition of happiness, and the consciousness of useful effort be the source of the highest earthly satisfaction, who is there at the renewal of the duties of a new year that may more appropriately indulge anticipations of happiness than the faithful votary of the healing art? Can he look back upon the year that is past with an approving conscience, satisfied that opportunities for personal improvement in professional knowledge and skill, and for the diffusion of their benefits to mankind, have been diligently improved,—then, a *happy new year* awaits him, albeit it will prove but a literal transcript of that which is ended.

But 'a truce with sermonising,' our readers, ere this, may have been ready to exclaim; 'have you no other *wishes* for New-Year's day,' it may be added, 'than that we poor doctors may contemplate our destiny with meek resignation?' In the customary enumeration of invocated blessings, on this occasion, health usually and properly takes precedence of all others. And health is signally of priceless moment to the physician. The nature of his duties requires his personal attention and activity. His offices cannot be delegated to another; nor can he repudiate the claims which are forced upon him with the eagerness of friendly partiality, and the urgent appeals of humanity. The indulgence which feeble physical powers, and mental exhaustion require, is seldom vouchsafed to him. He cannot fulfil the duty of repose which he may owe to himself. The melancholy fact of his own weakness and sufferings must often be carefully concealed. His task is to comfort and encourage others, and he well knows that without the influence of a cheerful countenance, and a seeming buoyant spirit, the resources of the *materia medica* are often unavailing. His experience sometimes is not unlike that of the comic player, who, amidst pain of body, or anguish of heart, is forced to appear the impersonation of humor. Well may it be a fervent wish for the medical practitioner that he may himself be spared the trial of disease.

All other temporal blessings are expressed by the word *prosperity* — a term of comprehensive scope, and indefinite signification. Were the imagination to represent all desires embraced in this word, what a medley of impracticabilities would be presented! Were that which is most desired to be attained by the act of wishing, how unsatisfactory often would be its possession! How dangerous would be the exercise of wishing, if it were not fruitless in its actual results; for what would be most wished for as blessings, might prove dire calamities! But, we are again moralizing; and since we are fairly committed to the train of thought which we have discursively pursued, we know of no remedy, but to lay aside our pen. Before we leave our editorial chair, however, we would speak a single word of comfort to one class of our readers. There are some who receive our monthly offering, whose professional career has not yet extended over many anniversaries of this description. The remark may perhaps be pardoned that we are not so far beyond the experiences of early medical practice, to have lost a vivid sense of its many anxieties, trials and discouragements. In view of these reminiscences we would tender to our junior brethren sympathy and encouragement. Courage, young friends! A brave heart and manly fortitude must in the end enable you to surmount every obstacle. Patience, perseverance and steady uprightness are the conditions of success, and these conditions fulfilled, success, if we may judge from the examples of those who have gone before us, will in the end be the crowning reward of diligence and fidelity. Holding fast to good principles, and high aspirations, each New-Year's day will bring nearer the goal of honorable wishes.

